# Iron Metabolism in Prostate Cancer; From Basic Science to New Therapeutic Strategies

**DOI:** 10.3389/fonc.2018.00547

**Published:** 2018-11-27

**Authors:** Driton Vela

**Affiliations:** Department of Physiology, University of Prishtina, Prishtina, Kosovo

**Keywords:** prostate cancer, iron metabolism, transferrin receptor 1, iron responsive element-binding protein 2, ferroportin, hepcidin, tumor associated macrophages, cancer stem cells

## Abstract

An increasing amount of research has recently strengthened the case for the existence of iron dysmetabolism in prostate cancer. It is characterized with a wide array of differential expression of iron-related proteins compared to normal cells. These proteins control iron entry, cellular iron distribution but also iron exit from prostate cells. Iron dysmetabolism is not an exclusive feature of prostate cancer cells, but it is observed in other cells of the tumor microenvironment. Disrupting the machinery that secures iron for prostate cancer cells can retard tumor growth and its invasive potential. This review unveils the current understanding of the ways that prostate cancer cells secure iron in the tumor milieu and how can we exploit this knowledge for therapeutic purposes.

## Introduction

Cancer cells are known for their voracious appetites for different metabolites and nutrients in order to satisfy their metabolic needs (1). Creating an environment that suits these needs means that cancer cells have to adapt manifold; they have to be able to secure energy for their metabolism in hypoxic conditions, but also they should be able to increase the transport of metabolites and micronutrients, even by “stealing” from their “neighborhood” (1). Eventually, this strategy proves successful for cancer cells, because they will invade transport fluids (blood, lymph) and spread across the body. One of the micronutrients that provides cancer cells the ability to grow and survive is iron. Its importance has been observed when cancer cells are introduced in iron-rich and iron-starving conditions. Iron-rich supply helps cancer cell growth, while iron starvation retards their growth (2, 3). But, cancer cells face a problem due to their high cellular iron supply; iron overload can cause an increase in reactive oxygen species, which are harmful for cell structures. Cancer cells have developed mechanisms that protect them against oxidative damage. These mechanisms are used by different cancers, including prostate cancer (PCa) cells. The increased activity of enzymes with antioxidant properties ensures a proper environment for cancer cells, where they can use high levels of iron without the accompanying side effect of oxidative damage (4–6). Thus, only very high levels of cellular iron can be detrimental for cancer cells (7).

Iron is used by cancer cells for important biochemical reactions, such as DNA synthesis, mitochondrial metabolism, tumor proliferation through increased angiogenesis, and metastasis (8). In PCa, iron is also important for tumor proliferation. Similar to other cancers, PCa cells need iron for their survival, including the use of iron for the activity of enzymes that control androgen receptor (AR) transcriptional activity, which is a known promoter of PCa (9). What is more, iron is needed by PCa cells to “reorganize” the intracellular enzymatic activity in order to increase energy production and extracellular matrix degradation (10, 11). Clinical data suggest that, in PCa, there is an increased iron sequestration in cancer cells, while in normal cells adjacent to PCa cells iron levels are low (12, 13).

## Iron entry in PCa cells

Transferrin receptors (TFRs) are major routes of transferrin-bound iron (TBI) into cells (14). TFR is responsible for endocytosing TBI and releasing it in intracellular compartments (14). Inside endosomes created during this process iron is reduced and finally released in cytoplasm via divalent metal transporter 1 (DMT1) (14). PCa cells need more TFRs to increase their iron uptake, which is why we observe upregulation of these receptors in this cancer (15–19). But, studies with cultured PCa cells have shown that iron can enter PCa cells even when TFR is blocked (10). PCa models show that TFR gene (TFRC) is a downstream target of the prostatic oncogene MYC (18, 19), while TFRC expression has been used successfully to detect precancerous prostate lesions with transferrin-based PET imaging (19). The dysregulation of TFR expression is not the only anomaly detected during TBI uptake in PCa cells. Endocytosed TFR is not localized in perinuclear sites in PCa (as it happens in non-tumorous prostate cells), but is rather distributed diffusely in the cytoplasm and cellular extensions (17). This could indicate that intracellular TFR traffic is another strategy used by PCa cells to redistribute iron for their metabolic needs (17). TFR has also been linked with AR gene expression, which is the main promoter of PCa. This link is at least partially mediated via the activity of vacuolar ATPase (V-ATPase), which is crucial in maintaining low endosomal pH (20–22). Low endosomal pH ensures the release of iron from transferrin, but when V-ATPase is inhibited less iron will be released from the cytoplasm (20). Low intracellular iron conditions increase the stability of hypoxia-inducible factor 1 alpha (HIF-1α), which in turn downregulates AR expression. This is important since AR downregulation retards PCa cell growth. Therefore, V-ATPase suppression has been proposed as an important target in PCa because it can inhibit tumor growth in different cancer lines, even in those resistant to androgen ablation (21). But, the activity of currently used compounds for suppression of V-ATPase is not cell specific which is why they are responsible for serious side effects observed in human patients (23).

Other important molecules studied in PCa are six-transmembrane epithelial antigen of prostate (STEAP) proteins. They are expressed in prostate tissue, where they serve numerous physiologic functions. STEAP2 is one of the most interesting proteins of the STEAP family known for its iron reductive properties (24). It is predominantly expressed in prostate tissue, located in the plasma membrane, but also in the intracellular vesicular structures (25). STEAP2 is frequently upregulated during prostatic malignancy, and importantly, its expression is in relation with Gleason score (24–26). Knockdown of STEAP2 significantly suppresses the proliferation of prostatic cancer, though current data suggest that this effect occurs probably due to STEAP2 ability to induce enzymatic activity that results in degradation of extracellular matrix (24, 27). The ability of STEAP2 to influence iron metabolism in PCa has not been studied, though the role of STEAP2 in iron metabolism has been observed in erythroid cells, choroid plexus, and gastrointestinal tract (28). On the other hand, intracellular six transmembrane prostate protein 2 (STAMP2, also known as STEAP4), which is another protein with metalloreductase activity, has been linked with PCa growth through dysregulation of iron metabolism (29, 30). Furthermore, it has to be noted that STAMP2 activity as a metalloreductase is more prominent compared to other STAMPs (31). Also, STAMP2 is regulated by AR, and similarly to STEAP2, its expression is linked with Gleason score (30). In other cancers, STAMP2 is involved in iron transport to mitochondria, but this action of STAMP2 has not been studied in PCa (32).

## Intracellular iron protein machinery in PCa

The most important intracellular regulators of iron metabolism are iron responsive element-binding proteins (IRPs). IRPs regulate the expression of iron import and export proteins (14). In low iron concentrations IRPs stimulate TFR1 upregulation and ferritin downregulation which ensures increased availability of iron for cellular needs (33). Although IRPs can modulate FPN activity as well, the existence of IRP-insensitive isoforms of FPN and of other more potent modulators of FPN make IRPs action on FPN modest compared to its actions on TFR1 and ferritin (34). Indeed, this observation has been confirmed in cancer cells as well (35).

**Figure 1:**
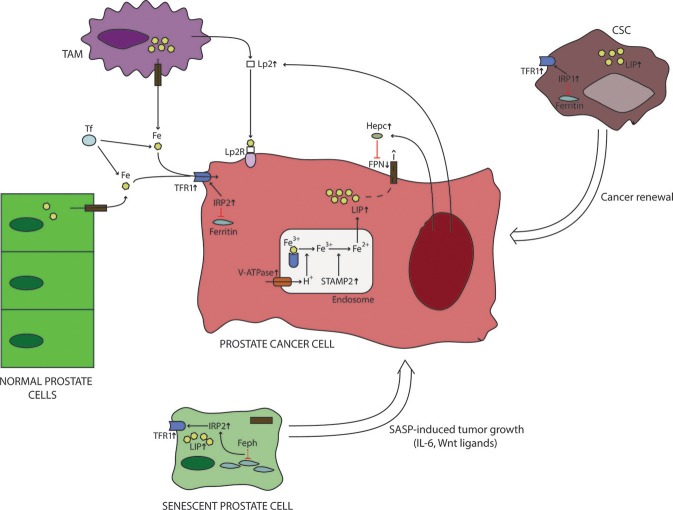
Iron metabolism in prostate cancer microenvironment. Prostate cancer cells are characterized with a differential expression of iron-related proteins compared to normal cells. These changes include upregulation of iron-import proteins (TFR1), overexpression of intracellular regulators of iron metabolism (IRP2), downregulation of iron export (FPN). In addition, recent data suggest that prostate cancer cells increase their labile iron pool by increasing the activity of hydrogen pumps and ferrireductases, which causes increased release of iron from endosomes. These actions will further increase labile iron pool. In addition, prostate cancer cells interact with their surroundings to increase iron delivery. This is believed to be done by stimulating TAMs to produce Lp2, which then binds iron and delivers it to cancer cells. Also, PCa cells secrete hepcidin locally to reduce their iron export through FPN downregulation and Lp2 to increase iron supply. Iron dymetabolism is also a feature of CSCs and sensescent prostate cells, whose numbers are increased in cancer. It is believed that iron dysregulation observed in CSCs is important for their survival and renewal of cancer cells. On the other hand, senescent prostate cells are known to secrete different molecules, some of which can directly influence the production of iron-related proteins by prostate cancer cells. CSC, cancer stem cell; Feph, ferritinophagy; FPN, ferroportin; Hepc, hepcidin; IL-6, interleukin-6; IRP, iron responsive element-binding protein; LIP, labile iron pool; Lp2R, lipocalin 2 receptor; SASP, senescence-associated secretory phenotype; STAMP2, six transmembrane prostate protein 2; TAM, tumor-associated macrophage; TFR1, transferrin receptor 1; V-ATPase, vacuolar ATPase; Wnt, wingless/integrated pathway.

In PCa IRP2 is upregulated to ensure proper amount of iron entry in PCa cells (16) (Figure 1). This effect occurs due to TFR1 upregulation and ferritin downregulation caused by IRP2 (16). The importance of IRP2 in PCa is observed during IRP2 knockout; downregulation of IRP2 significantly reduces proliferation of PCa cells in a similar fashion to iron chelation (16). On the other hand, loss of IRP1 does not seem to have profound implications on PCa iron metabolism and tumor growth (16). IRP2 dysregulation seems to be the norm in other cancers as well, where the consequences of IRP2 overexpression are similar to PCa (3, 36, 37). It is still not known what is the cause behind overexpression of IRP2 in PCa, but oncogenes are supposed to be potential culprits (16, 35).

## Iron export in PCa cells

Ferroportin (FPN) is the only known cellular iron exporter. In many cancers its activity is downregulated, which increases iron pool in cancer cells (38–40). Similarly, FPN dowregulation has also been observed in PCa (41–44). By stimulating FPN activity we can curb the growth and proliferation of PCa (41–45). This occurs due to cellular iron deprivation caused by increased activity of FPN. Furthermore, consistent FPN overexpression overrules compensatory mechanisms used by PCa cells to replete iron depots via increase in iron import (16, 44). More importantly, FPN overexpression reduces tumor proliferation in different types of PCa's, and irrespective of androgen sensitivity (44).

FPN expression is regulated by different stimuli. It is the main target protein of hepcidin, which induces FPN degradation (46). So, it is no surprise to find that local hepcidin is upregulated in cancer cells, including PCa (47–49). Prostatic hepcidin is regulated via interleukin 6 (IL6), similar to systemic (liver) hepcidin, but non-cannonical pathways, such as wingless/integrated (Wnt) pathway, sclerostin domain-containing protein 1 (SOSTDC1) and bone morphogenetic protein 4/7 (BMP4/7) are also responsible for PCa hepcidin expression, which suggests a differential control of hepcidin expression in PCa compared to liver hepcidin expression (47) (Table 1). Further evidence of involvement of hepcidin in PCa pathophysiology is its relation with prostate-specific antigen (PSA). In PCa cells with high hepcidin expression there exists a positive correlation between hepcidin and PSA expression, while markers of tumor proliferation and survival are significantly increased in PCa cells with hepcidin upregulation (48). In experiments with cultured PCa cells loss of hepcidin expression significantly reduces the proliferative ability of cancer cells, while addition of hepcidin increases the proliferative ability of cancer cell progressively (49). All these data support the notion that PCa growth can be retarded by influencing iron export through FPN. It is still not known how do other regulators of FPN expression, such as inflammation, assert their influence on FPN activity in PCa. The rationale exists, since inflammation has been shown to promote cellular iron accumulation in different cells, but also it can promote tumor proliferation in PCa (34, 50–52).

**Table 1:** Expression, regulation and effects of iron-related proteins in prostate cancer cells*.

**Iron-related protein**	**Expression**	**Regulation**	**Main effects**	**References**
TFR1	↑	MYC, IRP2	Increased iron import	(14–18)
IRP2	↑	Oncogenes (MYC?)	Increased iron import	(15)
Ferroportin	↓	Hepcidin, MZF1, Nrf2, ZNF217	Decreased iron export	(40–44, 47)
Hepcidin	↑	SOSTDC1, BMP4/7, IL6, Wnt pathway	Decreased iron export	(46–48)
STAMP2	↑	Androgen receptor	Increased reduction of Fe^3+^ to Fe^2+^	(28, 29)
V-ATPase	↑	Ac45 (V-ATPase associated accessory protein)	Increase in iron release from endosomes	(19, 21)

* *It includes only proteins whose activity has been shown to affect iron metabolism of prostate cancer cells. BMP4/7, bone morphogenetic protein 4/7; IL6, interleukin 6; IRP2, iron-responsive element-binding protein 2; MZF1, myeloid zinc-finger 1; Nrf2, nuclear transcription factor 2; SOSTDC1, sclerostin domain-containing protein 1; STAMP2, six transmembrane prostate protein 2; TFR1, transferrin receptor 1; V-ATPase, vacuolar ATPase; Wnt pathway, wingless/integrated pathway; ZNF217, zinc-figure protein 217*.

Another field of study with many unanswered questions is the relation of mitochondrial iron metabolism with PCa pathogenesis. The gene responsible for coding mitoferrin, which is a mitochondrial iron transporter, is upregulated in PCa patients, but the importance of mitoferrin in PCa cells has still not been elucidated despite its proposed role in cancer (53–56).

## Tumor-associated macrophages (TAMs) and cancer stem cells (CSCs) in PCa; new players influenced by iron metabolism in the complex PCa tumor environment

An often overlooked player in the tumor microenvironment are TAMs. They are leukocytes that promote tumor growth in different cancers, including PCa (57–61). Density of macrophages is associated with poor prognosis and more aggressive behavior of PCa (57–59). In PCa, TAM activity is influenced by chemical signals from tumor cells (59). One such signal could be the induction of lipocalin 2 (Lp2) secretion (62), which is why increased Lp2 from TAMs has been proposed as another strategy from cancer cells to provide additional iron for their needs (63). Lp2 is a protein which has a strong binding capacity for iron chelating molecules such as siderophores (64). It is produced by neutrophils, macrophages, epithelial cells but also by tumor cells (62–64). *In-vitro* and *in-vivo* data with breast cancer models show that iron-loaded Lp2 produced by TAMs can increase the iron pool of tumor cells, and consequently, influence their growth (64, 65). The importance of Lp2 in PCa iron metabolism is further enhanced by its frequently observed overexpression in PCa which is related with cancer proliferation (66–69). Furthermore, serum Lp2 has been found in high levels in patients with PCa (69). Still, the details of the TAM-Lip2 connection with PCa cell iron metabolism have not been studied and should be a subject of future studies.

In terms of their actions in tumor microenvironment macrophages have been generally divided into cytotoxic (M1 macrophages) and tumor promoting phenotype (M2 macrophages) (58). One of the most important population of leukocytes in cancer including PCa are M2 macrophages. Studies have unraveled the iron-release phenotype of these macrophages, which would support the proliferation of neighboring cancer cells (58, 59, 70–72). In addition, macrophages treated with iron chelators reduce the growth of tumors and their metastatic ability by changing their iron releasing phenotype into an iron sequestring one (71, 72). Similarly, in PCa, iron-laded macrophages exhibit infiltrative behavior and predict low response to iron chelation therapy (73).

Dysregulation of the iron metabolism is not an exclusive feature of cancer cells in PCa. Recently, it has been observed in CSCs of PCa as well (74). This is an important observation, because CSCs as precursors of cancer cells have been linked with the replenishment of cancer cell numbers, metastasis and resistance to cancer therapy (75). In models with cultured CSCs, iron supplementation helps cellular growth, while iron chelation retards it (76–78). More importantly, iron chelation has the benefit of having the impact on stemness markers of CSCs even when standard chemotherapy fails (76). In PCa, CSC labile iron pool is increased due to upregulation of iron import proteins. The intracellular regulators of iron metabolism in PCa CSCs show distinct features; IRP1 is upregulated in both mRNA and protein levels due to inefficient Fe-S cluster assembly in mitochondria, while IRP2 is not (74). It is interesting that while IRP2 is subject of regulatory control by iron levels in CSCs, the same does not occur in differentiated cancer cells (3, 16, 36, 74, 79). This suggests a possible scenario for progression of iron dysmetabolism in PCa; it involves IRP2 overexpression in differentiated cancer cells as an important moment when a full blown loss of the regulatory mechanisms occurs in cancer cells, which would create a vicious cycle of iron overload and resultant progressive proliferation of cancer cells. This might have important clinical implications for the treatment of PCa, and in other cancers as well.

## Iron dysmetabolism is a characteristic of aging prostate cells?

It is well-known that PCa is a disease of the old age, and similarly, prostate cell dysmetabolism could be an important pathogenic feature of aging prostate cells. It is known that the increased number of senescent cells is associated with age-related diseases (80). Recent data suggest that prostate senescent cells suffer from an increased iron load due to changes in the expression of proteins that regulate cellular iron transport (81). Iron dysregulation observed in experimental models with senescent prostate epithelial cells is intriguing and strikingly similar to PCa cells; it is characterized with TFR1, IRP2, ferritin upregulation, while FPN though upregulated, is mostly localized intracellularly, which means that it cannot participate in iron export (81). The reason behind these changes in senescent cells occur due to impaired ferritinophagy, which means that increased iron is sequestered into ferritin. This would signal senescent cells that there is a lack of intracellular iron, which in response increase iron import through TFR1 upregulation. Chemical induction of ferritinophagy reverses this process (81). Future studies should investigate potential disturbances of the process of ferritinophagy and its role in PCa.

Senescent cells accumulate in cancer and contribute to the pool of cancer cells by releasing chemicals that promote tumor growth (82). Some of these chemicals, such as Wnt ligands and IL-6, are known as upregulators of hepcidin in PCa cells (47, 83). On the other hand, it remains to be seen if the observed iron phenotype of prostate senescent cells affects its secretome and subsequent tumor growth.

## Systemic iron metabolism and PCa

Clinical studies suggest that homozygote carriers of hemochromatosis mutations are not especially in risk for PCa (84, 85). Iron has also been ruled out as an important occupational risk factor which could influence PCa occurrence (86, 87). Similarly, dietary iron intake does not seem to increase the risk for PCa, though there are doubts that iron intake might be associated with aggressive forms of PCa, especially in men with low intake of antioxidant foods (88, 89). On the other hand, data from a Dutch cohort with PCa patients showed that the combined intake of oxidants (iron) and antioxidants was not related with risk of advanced PCa (90). The Dutch cohort registered heme iron intake, while CARET cohort registered total iron intake, which might have been higher compared to their European counterparts. In any case, the relevance of Dutch cohort compared to CARET cohort is strengthened by a much higher number of patients included in the Dutch study (88, 90). Also, the associations between high iron intake/low antioxidant intake and aggressive PCa observed by CARET were not strong not just due to a smaller number of patients but also due to the fact that the associated positive trend was not statistically significant. Finally, other factors might have influenced results from CARET cohort; PCa patients from CARET cohort had higher body mass index (BMI), which is important, since studies suggest that BMI values indicative of obesity are related with higher Gleason score (or more aggressive PCa) (91, 92). It is interesting to notice that blood donors and blood recipients are not significantly protected or at risk from PCa, which further strengthens the case that systemic iron overload is not a significant factor for PCa risk (93, 94).

Other serum markers of iron metabolism have been studied in PCa, albeit in studies with small number of patients. For example, high levels of sTFR and hepcidin have been observed in these patients (95–97). Increased levels of sTFR are to be expected, considering the reactive TFR upregulation in erythroid cells due to anemia (often found in patients with PCa) and the increased expression of TFR in PCa cells. But, larger studies with different group of patients characterized according to age, inflammatory status, anemia status, local TFR, sTFR, and their correlations should give more answers concerning the importance of sTFR in PCa. On the other hand, one small study has revealed that serum hepcidin is increased in patients with bone metastasis, and that the increase in serum hepcidin is partly related to cytokine production, such as IL6, which is a known upregulator of hepcidin (97). In any case, these studies are rare and little conclusions can be taken out of them.

More contradictory data come from studies relating ferritin levels and its association with PCa. High and low levels of serum ferritin have been associated with PCa risk, while others have suggested no such association (96, 98–100). The differences in patient numbers and selection between the studies might have been the reason behind these discrepancies. Kuvibidilla et al. study, which reported presence of low levels of serum ferritin in PCa patients, included a small number of patients (100). In addition, the statistical significance was borderline and most of the patients did not have advanced PCa. It has to be mentioned that normal range of serum ferritin is very wide, therefore measurements of serum ferritin in a small number of patients, is subjected to false positive or negative results (101–103). Chua et al. study (which reported no association between serum ferritin and PCa risk) included a relatively low number of patients with PCa, and also did not have any information about the cancer stage of the patients (99). In one of the largest studies (2002 patients) of this kind done by Wang et al., serum ferritin was shown to be a predictor of PCa risk (98). Close examination of the Wang et al. study reveals that the differences in median values for serum ferritin though significant are still small. But, when the patients were grouped according to increased levels of ferritin, the statistical differences were increased, which means that the biggest odds ratio for PCa risk was detected with serum ferritin levels above 400 ng/ml. Also, this study revealed that the diagnostic sensitivity of serum ferritin in PCa was highest in patients with age >65 and serum ferritin >400 ng/ml. Finally, the study revealed that higher serum ferritin values were associated with higher Gleason score, and higher serum ferritin was associated with high PCa tissue ferritin. This is in-line with other studies that suggest that high serum ferritin is most likely observed in advanced PCa (96, 104). Wang et al. study has revealed that high serum ferritin is related with PCa risk, but only in a subset of patients with advanced PCa, and especially older patients. The increased serum ferritin in PCa could occur from different sources; cancer tissue, cytokines, damaged cells (105, 106).

## Treating PCa by modulating iron metabolism

The strategy to treat PCa by modulating tumor iron metabolism has already been subject of research. Iron chelators, first introduced in the 1960s, have been tested as anticancer drugs for 30 years (107, 108). In PCa, the use of experimental treatment with iron chelators goes back to 1995. In the study done by Kovar et al. deferoxamine (DFO) reduced tumor growth by 35 and 38%, respectively, depending on the tumor cell lines used (109). DFO efficacy was increased with co-treatment with anti-TFR antibodies. Soon after, first human trial was realized with a small number of patients suffering from hormone-refractory PCa (110). The use of DFO for 8 h did not yield any significant results in disease control. DFO failure in human trials occurred probably due to the drugs difficulty penetrating and accumulating in PCa cells when used in a systemic form (108). Deferiprone is another iron-chelator that has been used to treat PCa in cultured medium. It has shown antitumor activity in different PCa cell lines, by influencing tumor energy production, cell migration and tumor volume, but these results have not been validated in human trials with PCa patients (73, 111). Newer iron chelators have also shown antitumor properties but they have been only tested in cultured cells (112, 113). It has to be mentioned that iron chelation has its drawbacks, such as toxicity, patient compliance, induction of reactive increase in iron import proteins, lower efficacy with higher density of iron laded TAMs in tumors (73, 76, 107, 114).

Natural iron chelators have also been used for their antitumor properties. Curcumin, which is the active compound of the spice called turmeric, can suppress PCa growth. This action is attributed to curcumins ability to chelate iron, which is why PCa cells increase IRP activity and the expression of TFR1 in response to curcumin treatment (115, 116). Reactive response from tumor cells to curcumin actions can help them adapt to iron deprivation. This is probably the reason why TFR1 blockade in addition to curcumin supplementation improves the suppressive effect of this compound in PCa (116). It could be that the additional effect of curcumin in PCa is mediated by its inhibitory effect on hepcidin expression, which has already been observed in hepatocytes, but it has not been studied in PCa (117–119). Other compounds with iron chelating properties have been used in PCa cultured cells, such as epigallocatechin gallate (EGCG) (120). EGCG can retard tumor growth in PCa, but also suppresses PSA and AR expression (121–124). The effect of EGCG on PCa iron metabolism has yet to be studied but we know from its use in neurons that it can increase FPN activity and reduce hepcidin expression (125, 126). Adding to the evidence is the observation that frequent green tea use (EGCG is the main component of the green tea) has been associated with lower incidence of PCa (127). On the other hand, the effect of supplementation with different polyphenols, including EGCG and curcumin has been studied in a randomized trial with PCa patients. This trial has shown that supplementation which includes EGCG and turmeric can significantly reduce PSA levels in short-term (128). Unfortunately, this trial did not examine direct objective evidence (prostate biopsy, MRI) that would link more clearly supplementation with EGCG and curcumin with PCa progression. Use of natural compounds with the ability to modulate the iron metabolism in PCa cells has many challenges before they can be used as new drugs in treatment of PCa. One of the major challenges is the low bioavailability of the natural compounds (129, 130). Nanotechnology seems to come in handy when one wants to increase compound bioavailability. Sanna et al. developed nanoparticles encapsulating EGCG, which were able to increase the time to full degradation from 1 to 24 h (130). Another important property of these nanocarriers is the ability to have a high specificity for PCa cells. This was done by providing nanoparticles with ligands that are able to bind to one of the most frequently observed antigen in PCa cells, such as prostate specific membrane antigen (PSMA) (130). The use of nanoparticles induces the inhibition of cancer cell viability more potently compared to “normal” EGCG, in both, *in-vitro* and *in-vivo* experiments (130). Similar results were achieved with curcumin loaded nanoparticles (131, 132). In any case, it is pertinent for future studies to evaluate the concrete mechanistic model of action of EGCG and curcumin in PCa iron metabolism.

Recent data suggest that the antineoplastic arsenal of iron therapy in PCa is wide and with different available options. In addition to the already established antitumorous effect of local TFR1 blockade (109, 133), emerging data has revealed that local iron dysmetabolism can also be tackled by local suppression of hepcidin, IRP2, V-ATPase, STAMP2, and stimulation of FPN activity (16, 20, 30, 41, 42, 47) (Table 2). These results further strengthen the case for cellular iron deprivation therapy as a viable option in treatment of PCa.

**Table 2:** Therapeutic possibilities of the manipulation of iron metabolism in prostate cancer.

**Compound**	**Model of study**	**Mechanism of action**	**References**
Suramin	Prostate cancer cell cultures	Decreased iron import by blocking the binding of TF to TFR	(132)
Anti TFR antibody+deferoxamine	Prostate cancer cell cultures	Anti TFR antibody reduces iron import Deferoxamine acts via iron chelation	(108)
Anti TFR antibody+curcumin	Prostate cancer cell cultures	Anti TFR antibody reduces iron import Curcumin acts via iron chelation	(115)
Deferiprone	Prostate cancer cell cultures	Deferiprone acts via iron chelation	(110)
Deferiprone	*In-vivo* animal model with implanatable prostate cancer cell cultures	Deferiprone acts via iron chelation	(72)
DFO+Dp44mT	Prostate cancer cell cultures	DFO+Dp44mT act via iron chelation	(111)
Dp44mT	Prostate cancer cell cultures	HDp44mT acts via iron chelation	(112)
EGCG	Prostate cancer cell cultures	EGCG acts as a possible iron chelator	(119)
Anti IRP2 lentiviral shRNA	Prostate cancer cell cultures	Decrease of iron import	(15)
Nrf2	Prostate cancer cell cultures	Increase of iron export via FPN	(41)
Anti-ZNF217 siRNA	Prostate cancer cell cultures	Increase of iron export via FPN	(44)
Human FPN cDNA clone	Prostate cancer cell cultures	Increase of iron export	(43)
Human FPN and MZF1 plasmids	Prostate cancer cell cultures	Increase of iron export	(40)
Anti-hepcidin antibody	Prostate cancer cell cultures	Increase of iron export	(46)
Anti-hepcidin siRNA	Prostate cancer cell cultures	Increase of iron export	(48)
Anti-STAMP2 siRNA	Prostate cancer cell cultures	Decrease in reduction of Fe^3+^ to Fe^2+^	(29)
Concanamycin	Prostate cancer cell cultures	Decrease of intracellular iron release from endosomes via V-ATPase inhibition	(19)

*DFO, deferoxamine; Dp44mT, di-2-pyridylketone 4,4-dimethyl-3-thiosemicarbazone; EGCG, epigallocatechin gallate; FPN, ferroportin; MZF1, myeloid zinc-finger 1; Nrf2, nuclear transcription factor 2; shRNA, small hairpin RNA; siRNA, small interfering RNA; STAMP2, six transmembrane prostate protein 2; TFR, transferrin receptor; V-ATPase, vacuolar ATPase*.

## Conclusion

Iron dysmetabolism is a feature of different cancer cells. Accumulating data suggest a similar scenario for PCa cells as well. The picture that has unfolded shows how PCa cells are able to manipulate iron metabolism for their purposes. The main changes include the overexpression of iron import proteins (TFR1), intracellular regulators of iron import (IRP2), proton pumps that cause intracellular iron release, and ferrireductases such as STAMP2. In addition, the decrease of iron export (through FPN) is also an important strategy used by PCa cells to secure abundant iron for their needs. But, the full picture of the contribution of iron transport proteins remains unsolved. For example, the contribution of DMT1 in PCa is unknown, with data from tumor initiating cells showing no specific role of this protein in the iron transport of these cells (74). Another unexplained issue is the role of non-transferrin bound iron (NTBI) in PCa, especially during systemic iron load. On the other hand, the emerging role of zinc transporter proteins in the transport of NTBI in different cells does not seem to be translated in PCa cells, because most of these proteins are downregulated in prostate tumor cells, which probably serves as a protective strategy of prostate tumor cells against intracellular zinc accumulation (14, 134). The unknown role of DMT1 and probably irrelevant contribution of zinc transporters in iron transport begs the question how do PCa cells secure iron even when TFR is blocked? (11).

Recently, studies have revealed the increased complexity of the iron dysmetabolism in PCa cells, which indicates that cancer cell proliferation is related with the iron phenotype of other cells of the tumor microenvironment, such as TAMs and CSCs. TAMs are influenced directly by tumor cells and they show an iron-releasing phenotype which suits cancer cells. We do not know exactly how do TAMs influence the iron pool of PCa cells, but FPN and Lp2 seem to be obvious suspects. Furthermore, the study of iron dysmetabolism in TAMs has not been accompanied by examination of the role of inflammatory signaling in shaping iron metabolism of PCa cells, which should be a focus of future studies. This is important considering that inflammatory signals are known as upregulators of systemic and prostatic hepcidin.

Apart from PCa cells, TAMs and CSCs, other cells of the prostate tissue are important to understand temporal changes in iron metabolism in PCa. For example, senescent prostate epithelial cells show striking similarities with PCa cells in terms of the changes in the expression of iron-related proteins. This is important because senescent cells accumulate in cancer and contribute to the pool of cancer cells by releasing chemicals that promote tumor growth. Still, the role of the iron phenotype of prostate senescent cells in affecting tumor growth in PCa has yet to be observed considering the lack of studies in this area of research.

What about adjacent normal prostate cells, how do they behave in PCa microenvironment? Interestingly, *in-vivo* data show that normal cells adjacent to PCa cells suffer from iron deficiency, which may suggest that iron-hacking strategy could be one of the mechanisms used by prostate tumor cells to secure abundant iron (12, 13). How could this action occur is still unknown, though the logical target could be FPN of normal cells.

Clinical data with human patients show that iron dysmetabolism found in PCa cells is generally a local phenomenon not related to changes in systemic iron metabolism (90, 98). Details from these studies suggest that systemic iron load might be associated with PCa in a subset of older patients with high levels of ferritin, though it is not known if high iron load in these cases is of primary importance or it has a secondary role by aggravating the already dysregulated iron metabolism found in PCa cells. Bigger studies with a significant number of patients with different stages of PCa should explain important questions in this respect by examining the role of systemic hepcidin, sTFR, and other markers of global iron metabolism.

Finally, the importance of iron dysmetabolism for the survival of PCa cells has been tested in cultured cells. Results show that growth of PCa cells is suppressed when compounds that can affect iron metabolism of cancer cells accumulate in PCa cells. These results have been obtained with iron chelators, inhibitors of TFR1, IRP2, hepcidin, V-ATPase, STAMP2, but also with stimulators of FPN activity. But, the translation of these results from *in-vitro* conditions to human trials is associated with many challenges, which as recent data suggest, might be curbed with the use of nanocarriers that increase compound bioavailability and enable their cell specific delivery.

## Author contributions

The author confirms being the sole contributor of this work and has approved it for publication.

### Conflict of interest statement

The author declares that the research was conducted in the absence of any commercial or financial relationships that could be construed as a potential conflict of interest.
